# Letter from K. P. Moore, M. D., Macon, Ga.

**Published:** 1889-12

**Authors:** K. P. Moore

**Affiliations:** New York


*Atlanta Medical and Surgical Journal:*

I expect that many of your Georgia readers, like myself, do not know your New York correspondent; and, while we all enjoy reading his letters and find in them much to interest us, yet you know we appreciate a letter from a friend or acquaintance much more than from a stranger; likewise, we read a book with much more pleasure and satisfaction when we personally know the author. So, while I shall make no pretension to writing so pleasantly or so instructively, yet perhaps a letter from an old Georgia doctor may not be entirely uninteresting, at least to the profession of my own State.

I find the same pluck and push among New York's leading doctors which has always characterized them, and they have no disposition to keep their light under a bushel. Most of them go abroad during the summer months and catch up all that is new and progressive, and are ready, when the fall and winter come, to unload their artillery upon the hundreds of students and medical men who come to this great city seeking light.

The time has come, I am happy to say, when many of our best men are beginning to make themselves and their merits felt abroad, and the unloading is not unfrequently done "on the other side." It is now a recognized fact that many of our American authors are fully abreast, if not in advance, of those in the old world, and are becoming the moulders of opinion in many departments.

The day after my arrival in New York, I visited the Polyclinic, and was taken possession of by my friend, Dr. John Wyeth, the head and shoulders of this first-class school. I had the pleasure of taking lunch with him and his excellent family at their home on Fifth Avenue. The last time I was in New York I had the pleasure of meeting Mrs. Wyeth at her home, and while she was then "full of grace and beauty," and a visit to her home was a real pleasure, it has now been largely increased by the presence of two jolly and beautiful little ones, whose presence bring into the household no little sunshine and joy. Here I also had the pleasure of meeting and renewing the acquaintance of Dr. H. Marion Sims, the brother of Mrs. Wyeth, and, by the way, a "son of his father;" and while he will never fill the place made vacant by the death of his father (such men only come once in centuries), yet he is a *solid* young man, of real merit, and will make his imprint upon the pages of time.

In the afternoon, I went with Dr. Wyeth to Mt. Sinai Hospital, and saw him do some very fine bone surgery. As a surgeon he has but few equals in his age. He operates with boldness and fearlessness, and yet with due caution and conservatism. His operations are "clean cut" and thorough, and yet done with as much care and modesty as if done by a delicate woman. His work on surgery is a fine production, and has met with an immense sale. The second edition of it will be issued early in the coming year.

Dr. Sims also invited me to be present while he did Tait's operation the next day at Polyclinic Hospital. I was much delighted to see how easily and skilfully he did the operation, and the only complaint which I had to make against him was that he should even for once call it "Tait's operations." To this accusation he cheerfully owned up like a little man, and true to the opinion of his immortal father, he cheerfully accorded to our Battey all the glory. He said that in reality Tait did nothing more than Battey. I have had several tilts since I have been in New York in reference to the *name* of this operation. I am proud of Battey, and, as a true Georgian, I do not propose to stand by and allow his thunder stolen without being heard from.

A case of considerable interest occurred a day or two since at the Polyclinic, which led to quite an interesting lecture from Professor Wyeth, reviewing the subject of anæsthesia, or rather I should say, the relative effects and uses of the two principal anæsthetics, chloroform and ether. I don't know how I could better or more profitably interest your readers than to report this case, and then give you a brief *résumé* of the lecture.

The case was an epithelioma of the lower lip. The entire lip had been removed eight months ago, and a plastic operation was done, in which, in closing up, a small fissure was left. It was to remove, this fissure that the present operation was done. The patient was put under the influence of ether, by inhalation, and as the operation was one on the mouth, the anæsthesia was continued per rectum.

The plan of giving it was very simple. About four ounces of ether was placed in a ten ounce graduated bottle, through the cork of which a small conducting tube pierced, and the bottle immersed in water, at a temperature of about one hundred and twenty degrees, and in this way the ether fumes were driven into the rectum. The operation was quickly and skilfully done, consuming not more than twenty minutes. Not exceeding a half ounce of ether was used per rectum. The patient came from under the anæsthesia as kindly and reaction was as satisfactory as one could wish. The operation was done in the amphitheater at the Polyclinic at 11 A. M., and all went well until 8 o'clock the same evening. During the afternoon, the patient was cheerful and happy, and complained of no special inconvenience. At 4 P. M. he complained of the least bit of chilliness and slight fullness in his belly. At 8 o'clock a diarrhœa supervened, which increased in severity up to midnight, but as the patient made no complaint of it up to this time, and as he went himself to the water-closet, there was nothing to attract any attention to him, and no one suspected that anything serious was going on. He now complained to the orderly, who found the man so ill that he thought it best to call the house surgeon. He rapidly went into collapse and died at 3 A. M. An autopsy was made the next day by Dr. Bennett S. Beach. Both lungs were slightly emphysematous, with some pleuritic adhesions, and hypostatic congestion at apex of left ; small quantity of fluid in pericardial sack ; valves normal ; *post mortem* clot in right ventricle ; liver large, firm and pale ; spleen normal ; kidneys congested, but capsules not adherent ; stomach empty, but much distended ; upper two-thirds of small intestines empty ; lower third mucous membrane intensely congested and partly filled with fluid resembling blood, and a few blood clots ; larger intestines distended with gas, and containing a quantity of bloody fluid, with mucous membrane intensely congested.

Now while this case and the whole proceeding was strictly legitimate and eminently proper and right, and the operation skilfully and scientifically done; and while every proper and possible precaution was used, and while it is true that no man under the sun could have anticipated the result, and no possible blame could attach to any one; yet, as Prof. Wyeth touchingly remarked in his lecture upon the subject, "there is moral in it which no thinking man will allow himself to overlook." We all know that it was not the *quantity* of ether that killed the patient; he came from its influence as happily as one could desire, and yet so small a quantity set up an acute enterior-colitis of so violent a character that hemorrhage and death followed in a few hours. Death, in this case, probably was due to the hemorrhage. The patient was constantly going to the water-closet from eight o'clock in the evening until he became completely exhausted. No one, not even the patient himself, knew that the hemorrhage was going on, and it was left for the autopsy to tell the sad tale. It does not take the tongue of a prophet to point out to us the lesson of *caution* which this case should teach us.

In discussing the relative virtues and uses of the various anæsthetics, Dr. Wyeth said that he did not now use the A. C. E. preparation any more; he did not know the exact chemical composition resulting from this combination, and hence he did not care to take any chances on it, especially when he had each one separately, and *did* know just what he had to deal with. Besides, his clinical experience with it had been a little unpleasant, and he had abandoned its use.

As to chloroform and ether, ruling out children and obstetrics, he would place the proportion of indications for the two as about ten per cent. for the former to ninety per cent. for the latter.

Included in the ten per cent. of cases in which chloroform should be given rather than ether, was, first, those in whom ether had been previously given and in whom it had acted badly—those in whom ether brought on rapid cyanosis and tendency to respiratory paralysis; second, those with an existing bronchitis or chronic broncho-pneumonia, or those with specific gumata or other material obstruction in the lungs; and thirdly, those with Bright's disease in any stage. Briefly stated, he would not give ether in bronchitis, pneumonitis or nephritis.

Professionally speaking, Dr. Wyeth said he had been born and raised a chloroformist; so that all his predilections and early training led him toward chloroform, and not until long experience, close observation and careful study of the subject had forced him, did he give up its use and place himself on the side of ether. He said that up to within the last five years chloroform was the anæsthetic used in Europe, Germany and on the Continent, since which time ether had been gradually gaining ground; and although chloroform was the principal anæsthetic now in use in England and Scotland, he was confident it was due to the fact that they did not know how to use ether. He read extracts from the late inaugural address of Mr. Teal as President of the British Medical Congress, pnblished in the *Medical Record* of New York, August 31st, 1889, in which this great surgeon made the confession that ignorance in the use of ether had long kept it in disrepute in Great Britian, and he made the prophecy that when *they* had learned to use it, it would become as popular with the European and Continental surgeons, as it was now with the American surgeons. Among the many objections which has always obtained against the use of ether was the length of time it has always taken to bring the patient under its influence, which not only entailed great loss of time upon the surgeon, but what was worse, increased the liability of shock and failure of the vital forces from prolongation of time to the patient, and the risk of setting up an acute bronchitis or pneumonia by spraying over the mucous membrane of the respiratory tracts the cold, etherized air. These objects had now been overcome by improved inhalers—the Clover Inhaler, for instance, which, by its construction, admitted only the minimum of air and by the rebreathing over and over again the expired air, not only economizes ether, but reduces the temperature of the ether vapor almost to the normal. With this inhaler the patient could be kept well anæsthetized with about one drachm of ether for every fifteen minutes.

Dr. Wyeth said that the tendency of ether was to paralyze the respiratory centers, and that of chloroform to paralyze the heart centers, and just as we appreciate the difference between a suspension of a respiratory process and the heart's action should we choose between the use of chloroform and ether. He quoted the trite expression of Hunter McGuire, that "when a patient died from heart failure he died forever." When the heart ceased to beat there was no power known among man to restore it again to action; while on the other hand, artificial respiration could keep the vital forces going for ten, fifteen or even thirty minutes after nature had ceased to exert herself, and the patient be thus brought back to life again. On the question of inhalers or the mode of administering ether, while he did not believe in the total exclusion of air, as was the tendency with some now, yet he believed in reducing it to the minimum, and that safety lies in the happy medium.

Personally, I have had occasion to abandon my first impression as to the use of chloroform. Twenty-one or two years ago, when I was a medical student, chloroform was used almost altogether; and although I have never had a large surgical work, yet I have used chloroform hundreds of times, and have rarely ever had an untoward symptom, and I have always felt that much of the bad results from the use of chloroform has been more from

*carelessness* than from the drug. If we would always suspend the chloroform when we had reached the point of *complete* anæsthesia, there would rarely ever occur any bad effects from its use. We should always seek to avoid going *beyond* this point, even with ether.

Our object shall have been accomplished when we shall have completely paralyzed the cerebro-spinal system of nerves; and whatever anæsthetic we may use, we should avoid bringing under its influence any of the involuntary organs; or those under the influence of the sympathetic system, the lungs, as well as the heart. That chloroform possesses a good many advantages over ether no one, I think, will deny; and if there were no more dangers accompanying its use, certainly all of us would prefer to use it. To be sure its potency will not admit as careless use of it as of ether; neither can we use laudanum with the same freedom as we do paragoric. But if from carelessness we do use more laudanum than is necessary and death ensues, let us not be so unjust as to lay all the blame at the door of the laudanum; yet I grant that if we can accomplish the same result with the paragoric, to carry out the figure, even though surrounded with delays and difficulties, we ought to do it. And if ether allows more latitude and affords a greater margin before reaching the danger point, I am in favor of ether. It is upon this ground alone that I have become a convert to the ether faith, and have for several years acted upon it.

You know, we American people are very much disposed, in our haste, to go into extremes. We also have a weakness to "run after strange gods," and to blindly follow our over-enthusiastic leaders; and on this question of anæsthetics I have thought that the ether preference was, like a great many things, a sort of "ether rage." And maybe our brethren "on the other side" may say that *we* on this side will use chloroform more when we "learn how." I have been for some years expecting to see the pendulum begin to swing back, and I am not sure now but that signs of it are beginning to be visible here.

I have found some first-class men here who are using chloroform more than ether, while others seem to be standing on the fence, ready to swing on to the tide whenever it begins to reflow. I may be a sort of slow coach, and during the next decade may again be left behind; but "at the present writing," having fallen into line, for the reasons already given, let us stick to the ether.

I came here more to study gynecology than anæsthetics, and feel that I have found some green pastures in this field. In my next, with your permission, I may devote myself to something in this direction; for the present, adieu. K. P. MOORE.

New York, Oct. 31st, 1889.

